# Efficient steam generation by inexpensive narrow gap evaporation device for solar applications

**DOI:** 10.1038/s41598-017-12152-6

**Published:** 2017-09-20

**Authors:** Matteo Morciano, Matteo Fasano, Uktam Salomov, Luigi Ventola, Eliodoro Chiavazzo, Pietro Asinari

**Affiliations:** Energy Department, Politecnico di Torino, Corso Duca degli Abruzzi 24, Torino, 10129 Italy

## Abstract

Technologies for solar steam generation with high performance can help solving critical societal issues such as water desalination or sterilization, especially in developing countries. Very recently, we have witnessed a rapidly growing interest in the scientific community proposing sunlight absorbers for direct conversion of liquid water into steam. While those solutions can possibly be of interest from the perspective of the involved novel materials, in this study we intend to demonstrate that efficient steam generation by solar source is mainly due to a combination of efficient solar absorption, capillary water feeding and narrow gap evaporation process, which can also be achieved through common materials. To this end, we report both numerical and experimental evidence that advanced nano-structured materials are not strictly necessary for performing sunlight driven water-to-vapor conversion at high efficiency (i.e. ≥85%) and relatively low optical concentration (≈10 suns). Coherently with the principles of frugal innovation, those results unveil that solar steam generation for desalination or sterilization purposes may be efficiently obtained by a clever selection and assembly of widespread and inexpensive materials.

## Introduction

Developing sustainable technologies is fundamental for mitigating the anthropogenic impact on environment^1^: global warming and clean water scarcity are progressively impacting our economies and societies^2–4^. Therefore, international collaborative efforts have taken place to limit global temperature rise (up to 2 °C respect to pre-industrial levels^5^) and address clean water scarcity in the most water-stressed areas, where nearly two-thirds of global population will live by the year 2025^6^.

Several filtration or distillation processes have been developed for the generation of fresh water from either brackish or sea water. Most of these processes are highly energy intensive and powered by fossil fuels^7^, whereas more sustainable alternatives have been recently investigated to couple clean water generation with renewable energy^8^.

Among these methods, solar distillation appears as one of the most economical and practical solutions for small scale desalination, particularly in remote and off-grid areas^8–11^. Steam generation by solar energy (solar steam) has been also recently investigated in a broad variety of other applications, for instance enhanced oil recovery^12,13^, power generation^14^, sterilization^15^, separation processes^16^ or evaporation-driven engines^17^. The present challenge in solar steam generation is to develop robust and cost-effective technologies with enhanced solar-to-vapor conversion efficiencies^18,19^.

Currently, large plants for steam generation from solar thermal energy rely on a cavity or surface absorbing solar radiation^20,21^, being the absorbed heat then used to evaporate water directly or by means of a carrier fluid. These solutions suffer from large surface heat and optical losses^18^; therefore, nanofluid volumetric receivers have been recently studied for increasing the solar-to-thermal energy conversion^22–25^. In case of installations with limited sizes, such as those for remote villages and small islands, solar steam can be also generated by inexpensive and easy to operate solar still technologies, where water is directly evaporated by solar radiation with generally low steam productivity^8,26^.

Since water evaporation is a surface process, only water molecules at the thin water-air interface can be driven into vapor phase by their energy state^27^. However, bulk heating of water leads to additional heat losses due to the energy transfer to the portion of water volume not directly evaporating. On the other hand, the localization of heat far from the evaporative surface may lead to boiling process, where thermal energy is also dissipated in non-evaporative portions of the fluid during the migration of bubbles to the water-air interface^28^. Therefore, several recent studies aimed to gather solar radiation solely at the water-air interface, in order to localize the temperature increase in the evaporative region thus achieving a more energy-efficient solar steam generation^27^. This strategy has been implemented thanks to nanoparticle suspensions or microporous structures.

A variety of nanoparticles immersed in aqueous solutions has been found to convert solar energy to steam with efficiencies up to 80%, being only 20% of the incident radiation lost to heating the liquid volume^16^. For instance, solvated gold nanoparticles (or nanoshells) under light illumination are able to efficiently convert light to heat by plasmonic effect^29,30^. Also carbon-based (e.g., graphene, carbon nanotubes, carbon black) or silicon nanoparticles have shown excellent broadband solar absorptance^31–33^. This localized temperature increase generates a vapor film at the particle-liquid interface, which tends to increase under continuous illumination, eventually coalescing with other nanobubble complexes^34^. Once a critical bubble size is achieved, the nanoparticle and its vapor envelope migrate to the water-air interface, where only vapor is released while the nanoparticle starts again this nonequilibrium steam generation^15,16^. Such evaporation process takes place with bulk fluid at temperatures far below the boiling point^35^, and it could be further enhanced by light scattering nanoparticles in the proximity of the water-air interface^36,37^.

On the other hand, solar steam can be efficiently generated (i.e., up to 85% efficiency^18^) at low optical concentrations by floating structures with mesoscopic porous materials and high light-to-heat performance. In this configuration, solar steam generation is promoted by the concurrent action of solar absorption, heat confinement in the evaporative region and capillary action^19^. The porous structure floats at the water-air interface and progressively pumps water by capillary effect to the surface exposed to light. Furthermore, the porous structure is made out of a thermal insulating material, in order to limit the temperature increase in the non-evaporative water volume. The structure is also expected to have excellent light absorption behavior. Taking inspiration from the seminal work by Ghasemi *et al*.^18^, several authors are now investigating novel materials with such thermal, optical and capillary properties. Ghasemi *et al*. synthesized a double-layered structure made out of exfoliated graphite (top, light absorption) and carbon foam or porous polymer skeleton (bottom, porous and thermal insulating support)^18,38,39^. Similarly, Ito *et al*. studied solar steam generation by a thin porous graphene sheet^19^, Zhang *et al*. by a polypyrrole coated stainless steel mesh^27^, whereas Liu *et al*. by a wood-graphene oxide composite^40^. Other studies, instead, reported free-floating plasmonic films of Au or Al nanoparticles^28,41,42^, black gold structures with nanoscale gaps^43^, double-layered thin films with top plasmonic layer^44^, textile-like paper substrates for Au film^45^, bimetallic hollow mesoporous plasmonic nanoshells^46^ or bifunctional porous structures with titania and gold nanoparticles^47^.

While the heat localization at the evaporative interface is fundamental to increase the solar-to-vapor conversion efficiency thus making solar steam economically viable, the commercial upscaling of this strategy by nanoparticle suspensions or porous solar-absorbing structures may be technologically challenging and, in our view, it should be carefully analyzed. In practical applications, the exposure to oxidative chemicals in water (e.g., hypochlorites) and air (e.g., ozone), other water contaminants and UV light may rapidly degrade the photothermal or the capillary properties of the nanostructured materials^27^, therefore shortening the service life of solar steam generators. Furthermore, direct solar vapor generation is particularly appealing for desalination or sterilization purposes in Third World countries, where–following the Jugaad innovation principles^48^–the need for high conversion efficiencies has to cope with widespread manufacturing techniques, durable materials, robust operations, easy maintenance and low capital costs. All these requirements are not necessarily met by advanced materials and, therefore, cost-efficient solutions for the steam production under one sun have been recently proposed by the authors^49,50^ and by other works in the literature^51–54^.

In this article, we set up and test both computationally and experimentally a simple solar steam generator, which is found to have comparable performances (up to roughly 87%) as those obtained by others who use instead advanced micro- and nano-structured materials. In contrast to previous works, the proposed solar steam generator can operate also under concentrated solar radiation, therefore allowing larger steam production rates and efficiency. While our device is competitive with nanoparticle- or structure-based solutions, the realized prototype has been manufactured with commercial inexpensive materials (e.g., copper, cotton, glass, polystyrene) and widespread mechanical machining. In fact, the device layout allows to absorb solar radiation and localize it in a narrow gap of evaporating water, therefore minimizing the energy losses related to heating the non-evaporative water volume.

## Results

### Solar steam generator

Similarly to the strategy suggested by Ghasemi *et al*.^18^ and other authors thereafter^19,27,28,38,41,43–47^, solar steam generation is here enhanced by the concurrent action of three phenomena: (i) heat confinement in the evaporative region; (ii) capillary action to distribute water in a narrow gap (iii) effective thermal insulation around the evaporative region. Here we recognized that the above conditions can be safely ensured during steam generation, relying upon common and inexpensive materials without resorting to advanced nano-structured materials.

**Figure 1:**
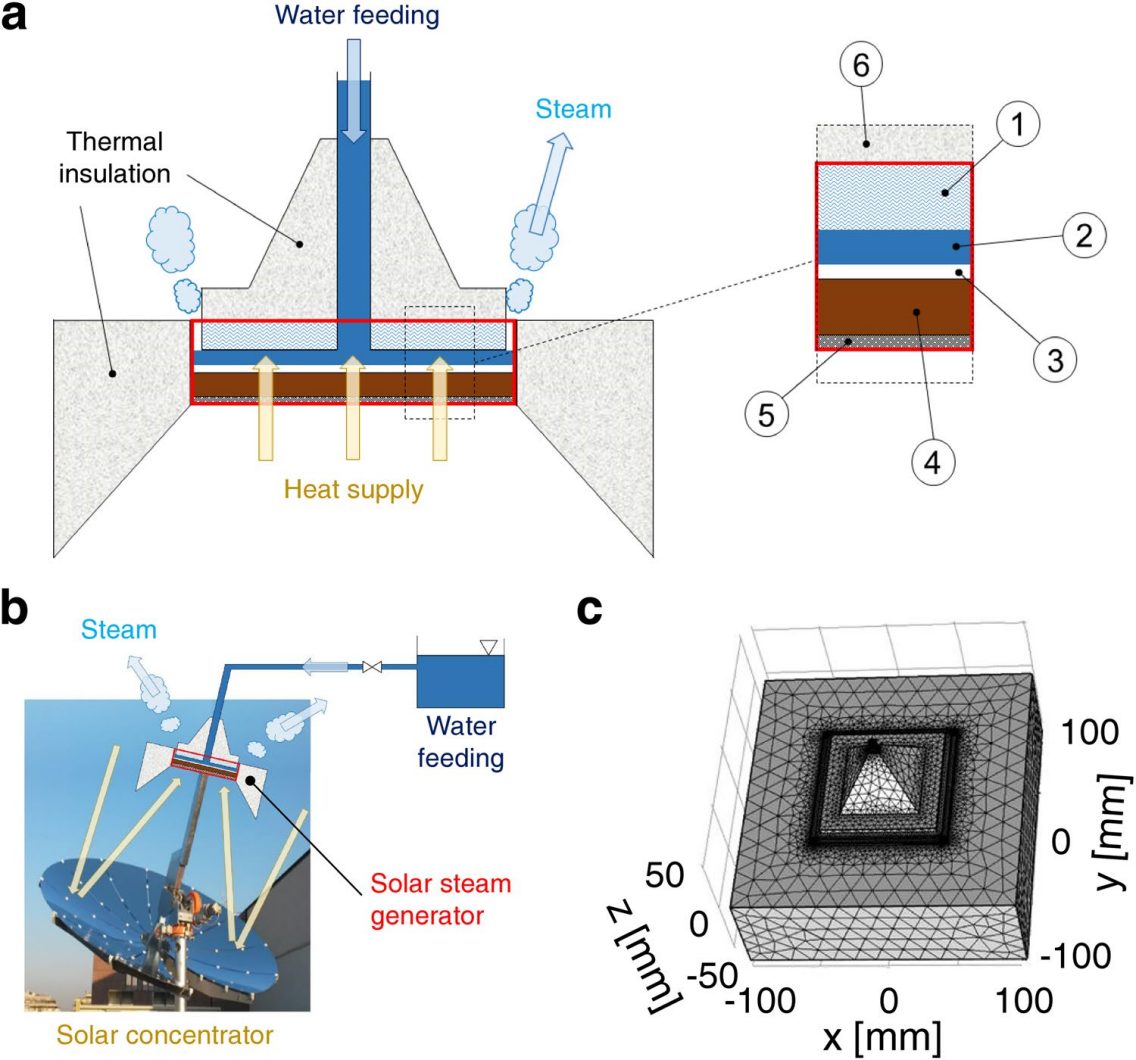
Solar steam generator. (**a**) Schematics and section of the solar steam generator: 1) glass; 2) narrow gap of evaporating water; 3) hydrophilic cotton; 4) copper plate; 5) commercial solar absorption material (e.g., TiNOX); 6) polystyrene. (**b**) Coupling between the steam generator and a solar concentrator. (**c**) Computational setup.

As sketched in Fig. 1a, the evaporation system is made out of a steam generator (red box), a water feeding system (blue arrow), a heat supply (yellow arrows), a tailored thermal insulation layer and a narrow gap for steam release (light blue arrows). Under operating conditions, the feeding system continuously provides water to the steam generator, through a pipe embedded in a upper conical element made of thermally insulating material. Water flows within the planar structure of the steam generator, and it can be driven to evaporation by thermal power coming for instance from a concentrated solar source. The steam generator mainly consists of a copper square plate (*S* = 9×9 cm^2^), a thin layer of hydrophilic material and a glass plate. Narrow gap (≈0.2 mm) evaporation takes place between the glass layer and the copper plate (Fig. 1a, inset). The bottom plate is made of copper because of its large thermal conductivity, which is needed for an efficient heat transfer from heat supply to evaporative region. The upper layer is made of glass, due to both its low thermal conductivity and hydrophilicity. Both the glass layer and a hydrophilic cotton bed on the copper plate promote a good water spreading by capillary action within the gap. Thermal insulation is also provided by a polystyrene envelope, whose shape and thickness have been carefully optimized by computer simulations.

Figure 1b depicts how the steam generator can be possibly coupled to a parabolic solar concentrator to produce solar steam. The steam generator can be readily placed in the parabolic dish focus, where solar radiation is concentrated on the external surface of the steam generator. The copper plate can be coated by off-the-shelf solar absorption materials (e.g., TiNOX^55^), which are characterized by excellent optical absorption properties and selective emission of radiation. This allows to efficiently convert the concentrated solar radiation into thermal energy, and to drive the narrow gap evaporation within the steam generator.

### Device performance

Four energy loss sources can be listed for the considered solar steam generator: (i) optical concentration losses; (ii) reflected sunlight by the solar absorbing surface; (iii) thermal conduction, convection and (iv) thermal radiation between the device and external environment.

While optical losses should be determined case-by-case according to the adopted solar concentration technology, the reflection of solar radiation is taken into account by considering the typical reflection losses by off-the-shelf solar absorption materials (${\eta }_{r}=0.95$ ^55^). Finite elements simulations are instead performed to detail the intrinsic thermal losses and thus evaporation efficiency of the steam generator. The steam generator depicted in Fig. 1a is first reconstructed by 3D modeling and properly meshed (Fig. 1c); then, several input thermal powers are imposed and thermal losses monitored. In detail, the evaporation performance of the steam generator has been evaluated in the range ${P}_{c}={\rm{\Phi }}/S=2\mbox{--}10$ kW m^−2^, namely 2–10 simulated optical concentrations (suns).

**Figure 2:**
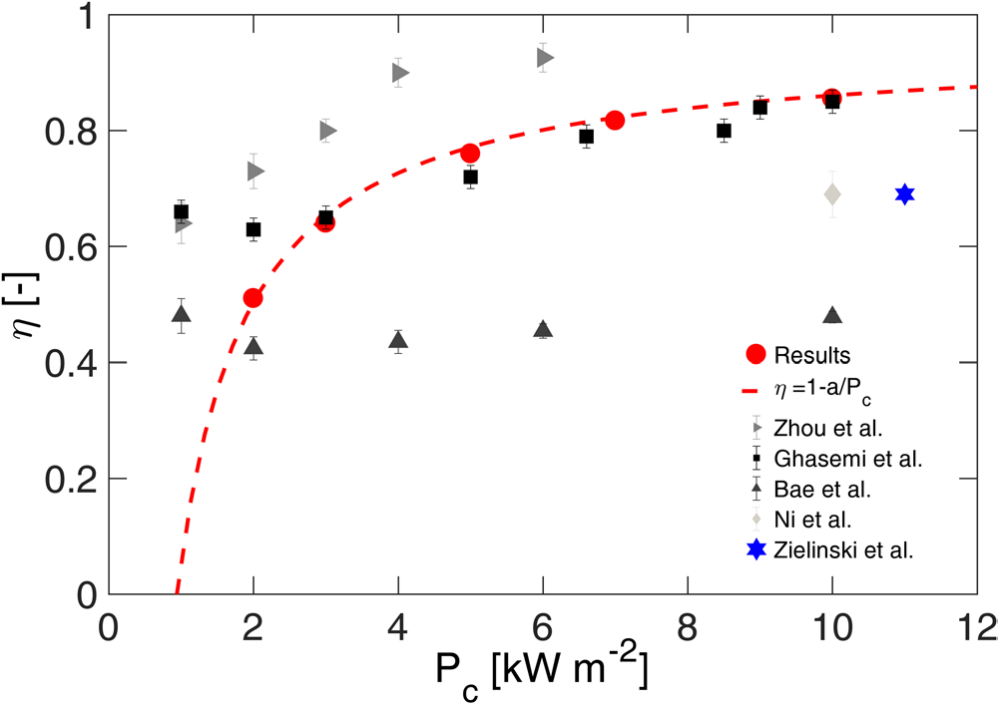
Evaporation efficiency. Evaporation efficiency of the solar steam generator, with different input thermal power (i.e., simulated optical concentration). Simulation results (red dots) are fitted by $\eta =1-a/{P}_{c}$ (red dashed line). Results are compared with the experiments reported by Zhou *et al*.^41^, Ghasemi *et al*.^18^, Bae *et al*.^43^, Ni *et al*.^31^, and Zielinski *et al*.^46^ (gray, black, dark gray, light gray, and blue symbols, respectively).

The overall efficiency of the solar steam generator can be then estimated as $\eta ={\eta }_{r}{\eta }_{e}$, where *η*_*e*_ is the evaporation efficiency and takes into account only the conductive, convective and radiative losses. Simulations show an evaporation efficiency *η*_*e*_ = 0.90 at *P*_*c*_ = 10 kW m^−2^, being radiation losses (≈3.5%) smaller than conductive ones (≈6.5%). The former are mainly due to the relatively large emissivity of the polystyrene coating (0.5–0.6), whereas the latter come mostly from convective heat losses from the bottom surface of the steam generator. The resulting overall efficiencies are plotted in Fig. 2 as a function of input thermal power (red dots). Note that optical concentration losses are not considered for a fair comparison with previous studies^18,31,41,43,46^. Results show a clear dependence of the overall efficiency with the input thermal power, namely the solar radiation concentrated on the solar absorption material coating the copper plate surface. In particular, *η* ranges from 0.51 (2 kW m^−2^) to 0.86 (10 kW m^−2^). It is worth noting that the solar steam generator performs best at high energy concentrations (i.e., over 5 suns), where efficiencies close to 0.80 are eventually attained; on the other hand, at lower solar concentrations, more than 50% of the input thermal power ends up in heat losses. This trend can be accurately (R^2^ = 0.99) described by $\eta =1-a/{P}_{c}$ (red dashed line in Fig. 2), where the best-fitted parameter $a=0.94$ kW m^−2^ represents the average thermal losses for the considered simulations.

These overall efficiencies can be successfully compared with the ones obtained by solar steam generators based on advanced materials. Figure 2 also shows the results obtained by Ghasemi *et al*.^18^ (black squares), where an exfoliated graphite structure with hydrophilic and interconnected pores is used to localize the solar radiation in the evaporative region^18^. Similarly to the current results, the solar steam generator by Ghasemi achieves 85% efficiency at 10 suns. On the other hand, it shows larger evaporation efficiencies at 1–2 suns, because of free surface evaporation phenomena (see Supplementary Note S1 and Fig. S1, for details)^56^. Figure 2 also presents the overall efficiency reported by Zhou *et al*.^41^ (gray triangles with right orientation), where over 90% efficiency is achieved at 4 suns by means of self-assembled gold nanoparticles on nanoporous templates. Instead, the evaporation efficiencies obtained by Bae *et al*.^43^ using black gold structures (dark gray triangles with upward orientation) are significantly lower than the current experiments, being *η* ≈ 50% at 10 suns. Finally, the light gray diamond and the blue star represent the performances obtained by Ni *et al*.^31^ (volumetric solar heating of nanofluids for direct vapor generation) and by Zielinski *et al*.^46^ (bimetallic hollow mesoporous plasmonic nanoshells), respectively: in both cases, *η* ≈ 70% at 10–11 suns are lower than the results found for the steam generator presented in this work.

### Experimental validation

The adopted simulation model accurately describes heat conduction in the interior of the steam generator as well as radiative and convective losses at its boundaries. All those phenomena determine thermal losses and are thus strictly sufficient to estimate the overall efficiency provided that evaporation stably occurs in the narrow gap of the generator. Therefore, the efficiency assessment reported in Fig. 2 also requires experimental tests aiming at confirming stability of water evaporation in our device.

**Figure 3:**
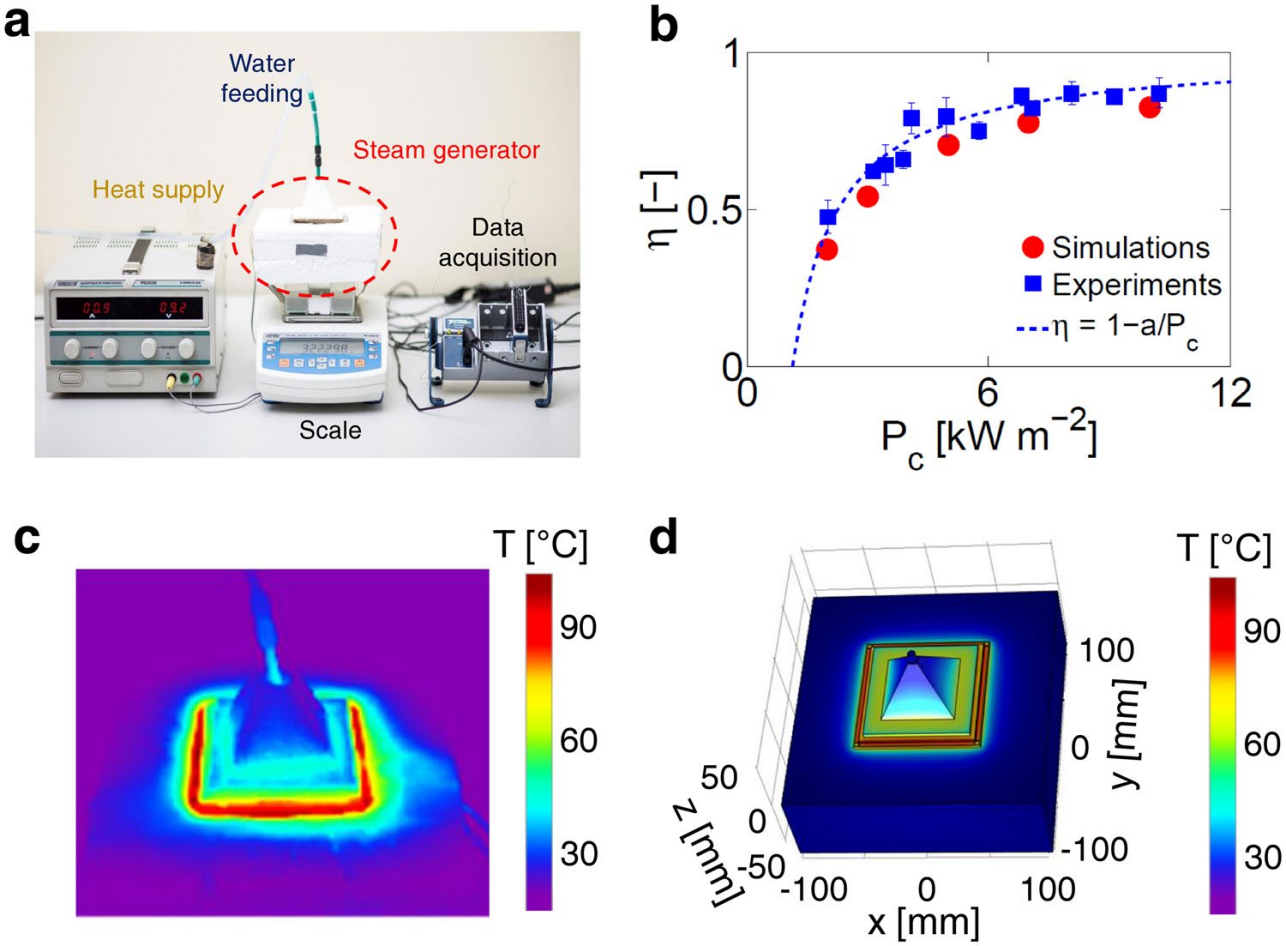
Lab-scale prototype. (**a**) Experimental setup. (**b**) Evaporation efficiency of the solar steam generator in experimental (blue dots) and simulated (red dots) conditions. Experimental results are fitted by $\eta =1-a/{P}_{c}$ (blue dashed line). (**c**) Temperature distribution in the steam generator under experimental conditions (${P}_{c}=10$ kW m^−2^), as obtained from thermal imaging camera. (**d**) Simulated temperature distribution (${P}_{c}=10$ kW m^−2^).

Figure 3a shows a lab-scale prototype reproducing the steam generator and the experimental setup adopted to evaluate its performances. As the main reason of experimental measurements was testing evaporation stability, for the sake of simplicity, the heat supply has been mock up by a tunable electric resistance, which is in direct contact with the copper plate and it is properly insulated by carton. This solution provided us with a better control of all operating conditions, therefore allowing an accurate evaluation of the steam generation process *per se*, without considering the optical losses due to concentration and the environmental variability of in-field testing. In our tests, water feeding is provided by a syringe pump, while scale and thermocouples allow to monitor device conditions (i.e., water mass and temperature) by means of a data acquisition platform.

The thermal losses of the prototyped steam generator have been estimated as a difference between input thermal power and steam generation. Several input thermal powers are tested in the experiments, to reproduce the steam generator performances under different solar concentrations. In each experiment, the ambient temperature lies in the range ${T}_{a}=21-24$ °C; while the temperature of the steam generator during the evaporation process (*T*_*c*_) is dictated by the phase change temperature. In all the experiments, stable evaporation processes are typically observed (see for example Supplementary Fig. S2).

Once evaporation rates and temperatures are measured at different *P*_*c*_, it is then possible to assess the evaporation efficiency of the steam generator as

1 $${\eta }_{e}=\frac{\dot{m}{h}_{LV}}{{P}_{c}S},$$

where $\dot{m}$ is the mass flow rate of generated steam and *h*_*LV*_ its total enthalpy change, which includes both latent and sensible heat. As a result, the overall efficiency (*η*, being *η*_*r*_ = 0.95^55^) ranges from 0.48 ± 0.05 (2 kW m^−2^) to 0.87 ± 0.05 (10 kW m^−2^). Again, this trend can be described as a function of the input power *P*_*c*_: $\eta =1-a/{P}_{c}$ (blue dashed line in Fig. 3b), where $a=1.13$ kW m^−2^ is the average thermal loss for the considered experimental setup.

Figure 3b allows to compare the efficiencies obtained by experiments (blue squares) with the ones calculated in the analogous simulation setup (red dots) at different *P*_*c*_. Results show an overall good agreement between experiments and simulations, in terms of both measured efficiencies and resulting temperature distribution, being experimental conditions (Fig. 3c) qualitatively confirmed by simulations (Fig. 3d). Note that the efficiencies calculated by simulations are generally lower than the experimental ones. In fact, while dynamic effects (e.g. boiling, turbulent mixing) may enhance the experimental evaporation rate, phase change is not directly modeled in the simulations, which therefore provide conservative performances of the steam generator. In this sense, simulations can effectively support the design optimization of the steam generator, aiming to further enhance its efficiency. For example, since surface radiation is the main thermal loss, covering the polystyrene surface by low emissivity materials (e.g., aluminum foils) may lead to a drop in radiative thermal losses, therefore improving *η*.

Note that passive strategies, such as capillary action, can be also adopted for the water feeding (see Supplementary Fig. S3, for details). In this configuration, the solar steam generator has been tested while floating on a water reservoir, from which water is continuously supplied through a hydrophilic channel by capillary effect. The input thermal energy can be provided by either direct or concentrated (e.g., by means of Fresnel lens) solar radiation. The floating steam generator fed by capillary effect has been prototyped and tested by mimicking the solar radiation with a electric resistance, with performances (*η* = 80% at *P*_*c*_ = 10 kW m^−2^) in good agreement with the results depicted in Fig. 3b.

### Evaporation process

**Figure 4:**
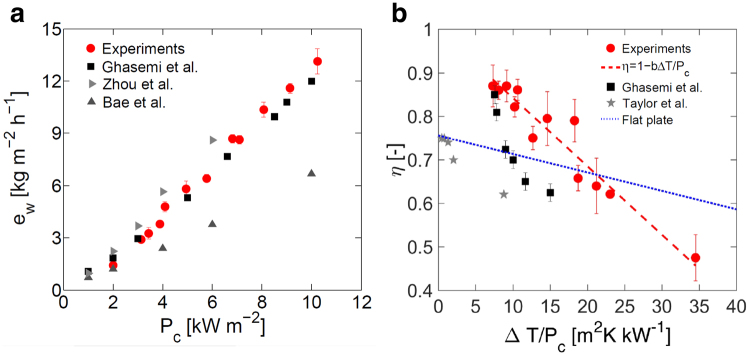
Evaporation performance and thermal losses. (**a**) Experimental evaporation rate of water (*e*_*w*_) in the solar steam generator, with different input thermal power (*P*_*c*_). Results are compared with the experiments reported by Ghasemi *et al*.^18^, Zhou *et al*.^41^ and Bae *et al*.^43^ (black, light gray and gray symbols). (**b**) Experimental evaporation efficiency of the steam generator plotted as a function of ${\rm{\Delta }}T/{P}_{c}$, where ${\rm{\Delta }}T=({T}_{c}-{T}_{a})$, *T*_*c*_ is the evaporation temperature and *T*_*a*_ the ambient temperature. Experimental points (red dots) are fitted by $\eta =1-b{\rm{\Delta }}T/{P}_{c}$ (red dashed line). Results are compared with results reported by Ghasemi *et al*.^18^ (black squares), Taylor *et al*.^57^ (gray stars) and commercial flat plates^58^ (blue line).

In Fig. 4a, the experimental evaporation rates of water measured in the steam generator are plotted as a function of the tested thermal powers (see Supplementary Fig. S2, for details). In detail, the prototype is able to produce up to 13 kg m^−2^ h^−1^ steam at 10 suns, namely a value close to the numerically predicted one (14.7 kg m^−2^ h^−1^). The exfoliated graphite structure designed by Ghasemi *et al*.^18^ shows a similar trend. Also the plasmonic absorber designed by Zhou *et al*.^41^ produces slightly larger mass flow rates, while the black gold structures developed by Bae and colleagues^43^ are characterized by lower steam productivities.

In Fig. 4b, the efficiency of the solar steam generator is also plotted as a function of the global thermal resistance ${\rm{\Delta }}T/{P}_{c}$, where ${\rm{\Delta }}T=({T}_{c}-{T}_{a})$ is the temperature difference between evaporation and ambient temperatures. The resulting trend can be well fitted (R^2^ = 0.92) by $\eta =1-b{\rm{\Delta }}T/{P}_{c}$ (red dashed line in Fig. 4b), being $b=15.75$ W m^−2^ K^−1^ a fitting parameter related to the average thermal performances of the device. Furthermore, in Fig. 4b the performances of the solar steam generator are successfully compared with the ones by Ghasemi *et al*.^18^ (black squares), state-of-the-art volumetric solar absorbers^57^ (light gray stars) and typical commercial flat plate collectors^58^ (blue line).

## Discussion

Developing more sustainable technologies for traditionally carbon-intensive applications is required for reducing the anthropogenic impact on environment. In particular, the generation of steam from solar energy has been extensively studied in several fields, among which desalination and sterilization. Recently, the efficiency of traditional solar distillation techniques has been significantly enhanced by concentrating the solar radiation solely at the water-air interface, in order to reduce the heat losses due to bulk heating of water in the non-evaporative regions. This solution has been implemented thanks to advanced materials (i.e., nanoparticle suspensions or microporous structures), which have been synthesized to guarantee high solar absorption, capillary effects and thermal insulation at the same time.

In this work, we demonstrate that the evaporation of water in a narrow gap, which allows to increase the solar steam generation efficiency, can be easily obtained by inexpensive materials and traditional fabrication processes (see Supplementary Note S2 and Fig. S4 for a comparative cost analysis of different solar steam technologies). In fact, a proper design of materials, shape and geometry of the steam generator is enough to achieve the concurrent solar absorptance, capillarity and thermal insulation obtained by advanced materials. The solar steam generator introduced in this work shows up to 87% solar steam generation efficiency at high energy concentrations (10 suns, optical losses not considered), thus even outperforming some of the solutions recently proposed in the literature. This proves that a proper design (e.g. based on the narrow gap idea) can greatly help in enhancing solar steam productivity without resorting to any advanced materials.

Most importantly, it is worth stressing that, despite the large solar-to-vapor conversion efficiency demonstrated in the several studies from the literature as well as the steam generator considered here, the productivity of clean water from steam condensation is still far from that obtained in large-scale conventional desalination plants. The main reason being that the latent heat of vaporization should be recovered and re-used to a large extent, for instance through multiple evaporation/condensation stages thus multiplying the quantity of treated water at a fixed solar input. Finally, the presented solar steam generator can possibly inspire new frugal innovations for low-cost solar-driven water desalination and sterilization, especially for remote areas in developing countries.

## Materials and Methods

### Simulations

Simulations of the steam generator performance are carried out by the finite element method implemented in COMSOL Multiphysics 5.0. The degrees of freedom of the problem are set to 190405 by tetrahedral meshing. Conduction (*k*, thermal conductivity), convection (*h*, convective heat transfer coefficient) and radiation (*ε*, emissivity) heat transfer mechanisms are considered in the simulations. The temperature in the narrow gap evaporation chamber is fixed at the evaporation temperature (*T*_*c*_), as experimentally measured. The ambient temperature is set to *T*_*a*_. The steam generator is made of polystyrene (*k* = 0.045 Wm^−1^ K^−1^), cotton (*k* = 0.04 Wm^−1^ K^−1^), copper (*k* = 400.0 Wm^−1^ K^−1^) and glass (*k* = 1.0 Wm^−1^ K^−1^). Convective heat transfer coefficients are estimated per each external surface of the steam generator, according to their temperature, inclination and geometry using well established empirical correlations from the literature^59,60^. More specifically, those natural convection coefficients range from *h* = 2.8 Wm^−2^ K^−1^ to *h* = 8 Wm^−2^ K^−1^. To evaluate radiative heat transfer, outer walls are assumed to behave like diffusive surfaces. The emissivity of TiNOX, a commercial material routinely used in thermal solar collectors with excellent optical absorption properties and selective emission of radiation, is taken as *ε* = 0.05^55^; whereas, polystyrene surfaces are characterized by relatively high emissivity (*ε* = 0.6).

### Experiments

The protocol for estimating the efficiency of the steam generator is made up of two steps, namely system equilibration and monitoring. The steam generator is first heated in dry conditions, until the evaporation temperature is achieved; then, water is cyclically introduced, being the injected mass and time interval between successive injections calibrated to achieve a stable and repeatable evaporation process. Once equilibrium conditions are reached, the evaporation rate is measured from the mass change rate of the initially injected water, whereas thermocouples are used to monitor the ambient and actual phase change temperature (e.g., see Supplementary Fig. S2). Hence, the reported measurements are limited to equilibrium conditions, while neglecting the initial transient. At least 10 evaporation cycles are considered to compute the average mass change rate (i.e., evaporation rate) and its standard deviation at fixed *P*_*c*_.

The steam generator characterized by laboratory experiments (Fig. 1) relies on the capillarity of hydrophilic cotton to homogeneously spread feed water through the narrow evaporative gap. In fact, a homogeneous distribution of feed water through the gap allows achieving a stable and efficient evaporation process, which would be instead deteriorated by the presence of dry regions (see Supplementary Note S3 and Fig. S5 for details). A syringe pump provides feed water to the steam generator by means of an on/off feedback control strategy (see Supplementary Fig. S2). When the temperature of the steam generator starts to increase (i.e., phase change is running out in some regions of the steam generator, which consequently starts to superheat), feed water is injected into the narrow gap to restore a homogeneous and stable evaporation process through the whole surface of the gap. The amount of injected water is dynamically dosed to avoid gap flooding, as clear by comparing Supplementary Fig. S2 a (3 suns, 3 kW m^−2^) and b (10 suns, 10 kW m^−2^). Note that, in principle, capillary effect by hydrophilic cotton would be also sufficient to guarantee a continuous evaporative process in the steam generator by only relying on a passive mechanism (i.e., without mechanical moving parts), as demonstrated by the floating configuration of the steam generator (see Supplementary Fig. S3).

## Electronic supplementary material

Supplementary Information
