# Supplementary material for: Efficient steam generation by inexpensive narrow gap evaporation device for solar applications
Source: Sci Rep. 2017 Sep 20;7:11970. doi: 10.1038/s41598-017-12152-6 (PMC5607388; doi:10.1038/s41598-017-12152-6)
Supplement: Supplementary file 1 — Supplementary Information [file 41598_2017_12152_MOESM1_ESM.pdf]

# Efficient steam generation by inexpensive narrow gap evaporation device for solar applications

**Matteo Morciano**[1,o], **Matteo Fasano**[1,o], **Uktam Salomov**[1], **Luigi Ventola**[1], **Eliodoro Chiavazzo**[1,*], **and Pietro Asinari**[1,*]

[1]Energy Department, Politecnico di Torino, Corso Duca degli Abruzzi 24, Torino, 10129, Italy
[*]Corresponding authors: pietro.asinari@polito.it and eliodoro.chiavazzo@polito.it
[o]These authors contributed equally to this work

# 1 Supplementary Results

**Note S1: Free surface evaporation** The efficiency of solar steam generator is lower at reduced power concentrations, as described by equation $\eta = 1 - a/P_c$ (see Fig. 2). On the other hand, in the experiments performed by Ghasemi *et al.*,[1] a plateau seems to take place at low power concentrations (i.e., less than 3 suns).

This phenomenon can be easily understood using classical models for free surface evaporation. While free surface evaporation cannot take place in the solar steam generator discussed in this article, the presence of a free water surface in the configuration by Ghasemi *et al.* induces an increased evaporation rate (≈30% more), thus evaporation efficiency, at low optical concentrations. For example, by considering the model by Shah:[2]

$$e_w = 35\rho_{s,wat}(\rho_{amb} - \rho_{s,wat})^{1/3}(x_{s,wat} - x_{amb}), \; [\mathrm{kg\ m^{-2}h^{-1}}] \tag{1}$$

where $e_w$ is the evaporation rate [kg m$^{-2}$h$^{-1}$], $\rho_{s,wat}$ is the density of saturated air (i.e., relative humidity $\varphi = 1$) at the water surface temperature, $\rho_{amb}$ is the density of air at ambient temperature and humidity (in the experiments performed by Ghasemi *et al.*, $\varphi = 0.31$ and $T = 24$°C), $x_{s,wat}$ is the humidity ratio of the saturated air (i.e., relative humidity $\varphi = 1$) at water surface temperature and $x_{amb}$ is the humidity ratio of air at ambient temperature and humidity.

The water surface temperature is critical to assess $e_w$. This value is not reported by Ghasemi *et al.*; therefore, we considered upper (steam temperature, see Fig. 5b in Ref.[1]) and lower (temperature of water just below the DLS, see Fig. S6 in Ref.[1]) bounds. In Supplementary Fig. S1, we compare the predicted evaporation rates by equation 1 with the experimental results by Ghasemi *et al.* at low power concentrations. The experimental results by Ghasemi *et al.* actually lie between the upper (gray dashed line) and lower (blue dashed line) bounds predicted by equation 1.

**Note S2: A cost estimate of steam production** A rough cost estimation of the recent technologies for solar steam generation is here performed, in order to compare the different solutions also from an economic point of view. Indeed, solar steam generation technologies are still at a very early stage, therefore costs could significantly decrease with industrialization; however, the following indications are reported to highlight that, despite similar technical performances, advanced materials may involve production costs orders of magnitude larger than solar steam generators made of traditional materials and machining. Here, the steam production cost of the prototype discussed in this article is compared for example to the ones estimated for the devices based on advanced materials described by Ghasemi *et al.*,[1] Ni *et al.*[3] and Zhou *et al.*[4] We stress that the estimates below are indicative, in the sense that they aim only to establish the order of magnitude of costs. These estimates are based on the best of our knowledge, available details reported in the literature and data-sheets from major suppliers.

Ghasemi *et al.* used a carbon foam structure in addition to exfoliated graphite to achieve increased evaporation performances. Considering the Sigma-Aldrich commercial catalog, the cost of a 150x150x2.5 mm$^3$ carbon foam structure is 187.5 EUR (`http://www.webcitation.org/6hQaZe0H9`), thus the cost for the volume of interest used by Ghasemi (50x50x10 mm$^3$) could be roughly estimated as 260 EUR. On the other hand, the cost of exfoliated graphite (82.10 EUR per 2.5 kg, `http://www.webcitation.org/6hQagagDJ`) can be neglected as compared to the carbon foam structure.

Ni *et al.*, instead, utilized nanofluids made of graphitized carbon black. Considering the Sigma-Aldrich commercial catalogue, the cost of 25 grams of nanofluid could be estimated as 142.5 EUR (`http://www.webcitation.org/6hQalLzAj`), which is determined by the mass fraction of nanoparticles solvated in the experimented nanofluid.

Zhou *et al.* exploited a plasmonic absorber, which consists of a golden thin film deposited on an alumina nanoporous template filled with gold nanoparticles. The cost of the nanoporous template is 150 USD/cm$^2$ (`http://www.webcitation.org/6hQasK83z`); therefore, by considering the area of the Zhou's device (≈ 6 cm$^2$), a cost of 900 USD can be roughly estimated. Furthermore, gold nanoparticles cost approximately 185 USD per 20 mL (`http://www.webcitation.org/6iFwTpTxC`). As a result, a final cost exceeding 1000 USD is reasonably expected for the whole solar steam generator. It is worth noticing that we have not taken into account the cost of the golden thin film deposited on the structure.

Concerning the lab-scale prototype tested in this work, we demonstrated that a stable narrow gap evaporation process can be achieved by only utilizing a copper plate (9x9 cm$^2$), hydrophilic cotton and a TiNOX energy foil (9x9 cm$^2$). The cost of the copper plate is in the range of 10-15 EUR (`http://www.webcitation.org/6iFxICcwc`); therefore, the cost of the whole prototype (including TiNOX energy foil, polystyrene insulation and glass layer) is about 25 EUR.

In Supplementary Fig. S4, the ratio between the cost of steam generators and the resulting evaporation rates (see Fig. 4a) is reported at different power density (i.e., 5 and 10 suns). By considering the cost per unit volume rate of produced steam, it is then possible to compare these technologies from an economic point of view. Note that, while previous studies already presented low-cost solutions for solar steam generation under 1 sun,[5,6] here we demonstrate for the first time that inexpensive but efficient solar steam technologies can be designed also for concentrated solar power conditions.

**Note S3: Functionality of hydrophilic cotton** The main functionality of hydrophilic cotton in the evaporative narrow gap of steam generator is to provide a better water spreading through the gap, in order to avoid dry regions deteriorating the overall

evaporation efficiency. Moreover, the experiments described in Supplementary Fig. S5 highlight that hydrophilic cotton has also the functionality to stabilize the phase change process in the narrow evaporative gap, therefore avoiding the formation of large vapor bubbles that lead to a lower steam generation efficiency. Finally, the large porosity of the thin bundles of cotton in the gap does not constitute a problem in providing an exit pathway to generated steam, which continuously flows out of the steam generator as demonstrated by the linear mass decrease in Supplementary Figs. S2 a and b.

Dry cotton is a thermal insulation material; however, the actual effective thermal conductivity of the narrow water+cotton gap in the steam generator can be roughly estimated as 0.45 $Wm^{-1}K^{-1}$, since it comes from a weighted average between the thermal conduction coefficient of cotton (0.04 $Wm^{-1}K^{-1}$) and water (0.60 $Wm^{-1}K^{-1}$). Hence, wet cotton presents a thermal conduction coefficient more than one order of magnitude higher than the dry one and, therefore, it does not represent a bottleneck in the heat conduction process from copper to fluid. As a perspective, metal foams with hydrophilic characteristics would be ideal candidates to replace the cotton layer, due to both large thermal conductivity and wicking properties.[7,8]

## 2 Supplementary Figures

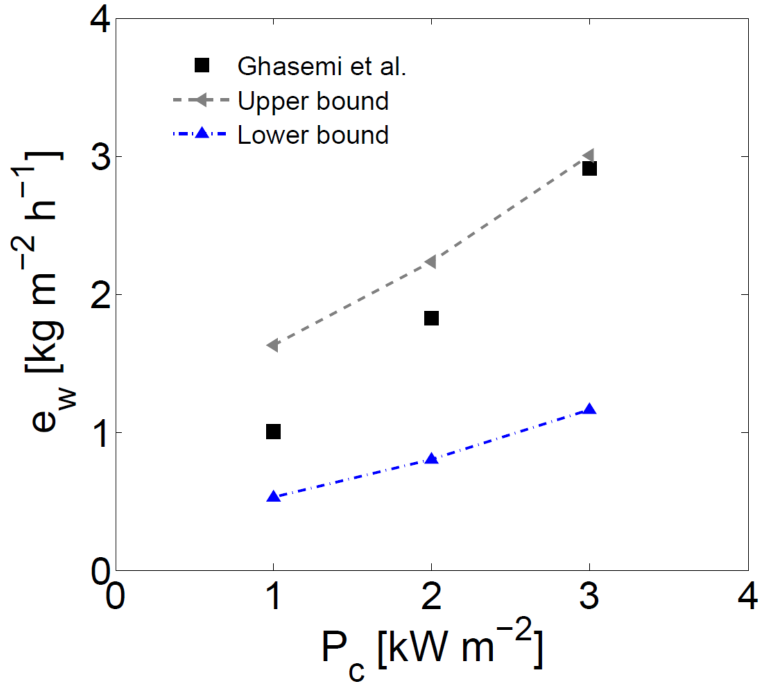


**Figure S1. Free surface evaporation estimates.** Experimental evaporation rates by Ghasemi *et al.*[1] (black squares) are compared with modeling predictions for free surface evaporation (equation 1). In the model, the temperature of surface water is considered as equal to the steam temperature (upper bound) or to the temperature of water just below the DLS (lower bound).

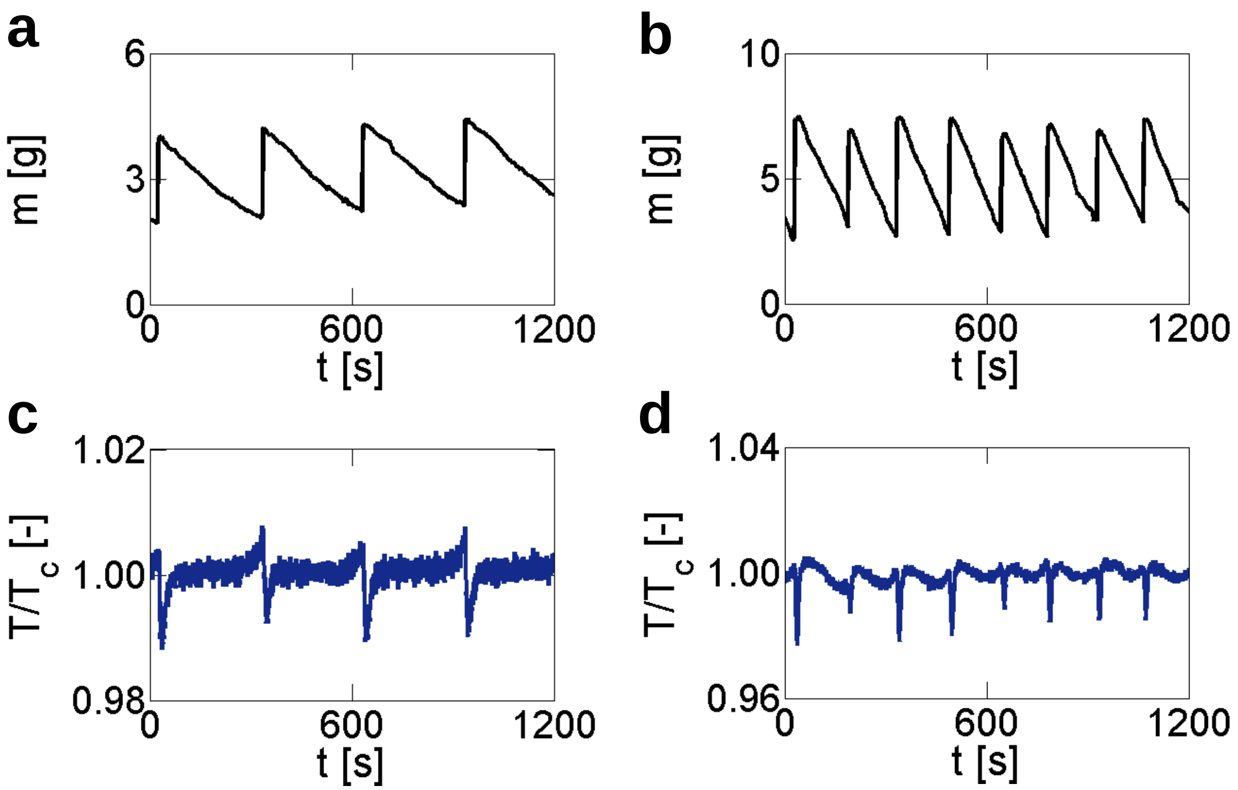


**Figure S2. Physical conditions in the experimental steam generator.** (**a**) Mass of liquid water within the steam generator, with $P_c = 3$ kW m$^{-2}$ or (**b**) $P_c = 10$ kW m$^{-2}$ input thermal power. By observing the different slopes in subplots a and b, it can be noticed that the evaporation rate $e_w = (dm/dt)/S$ is strongly dependent on the input power, being $S$ the surface where narrow gap evaporation occurs. For example, a 4.6-fold increase (from 2.88 to 13.13 kg m$^{-2}$h$^{-1}$) is recovered by varying the input power from $P_c = 3$ to 10 kW m$^{-2}$. (**c**) Temperature of the steam generator, with $P_c = 3$ kW m$^{-2}$ or (**d**) $P_c = 10$ kW m$^{-2}$. The average temperature of the steam generator ($T_c$) is 94.0 °C at $P_c = 3$ kW m$^{-2}$, whereas 97.3 °C at $P_c = 10$ kW m$^{-2}$.

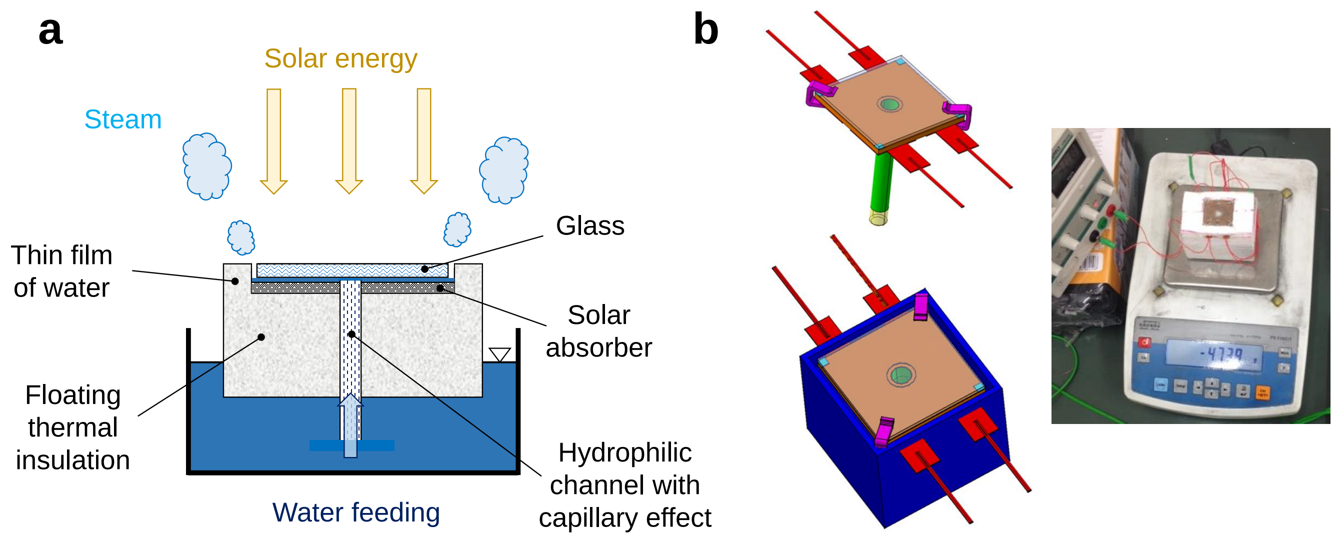


**Figure S3. Capillary water feeding.** (**a**) In this configuration, the solar steam generator floats on a water reservoir, from which water is continuously supplied through a hydrophilic channel by capillary effect. The input thermal energy can be provided by either direct or concentrated (e.g., by means of Fresnel lens) solar radiation. (**b**) The floating steam generator fed by capillary effect has been prototyped and tested by mimicking the solar radiation with a electric resistance.

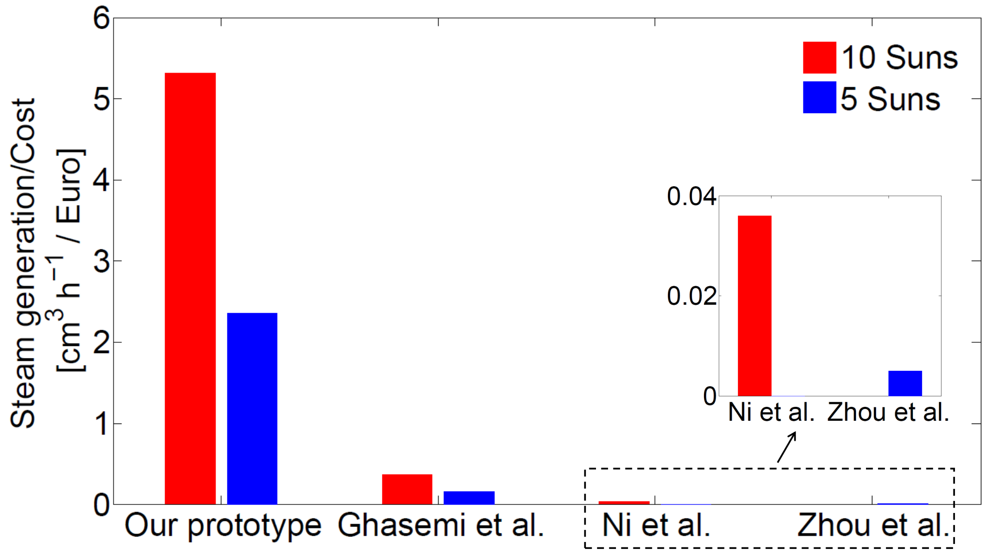


**Figure S4. Steam production cost.** The cost of steam generation obtained by our prototype, Ghasemi *et al.*,[1] Ni *et al.*[3] and Zhou *et al.*[4] are plotted at different power concentrations, namely 10 suns (red bars) and 5 suns (blue bars).

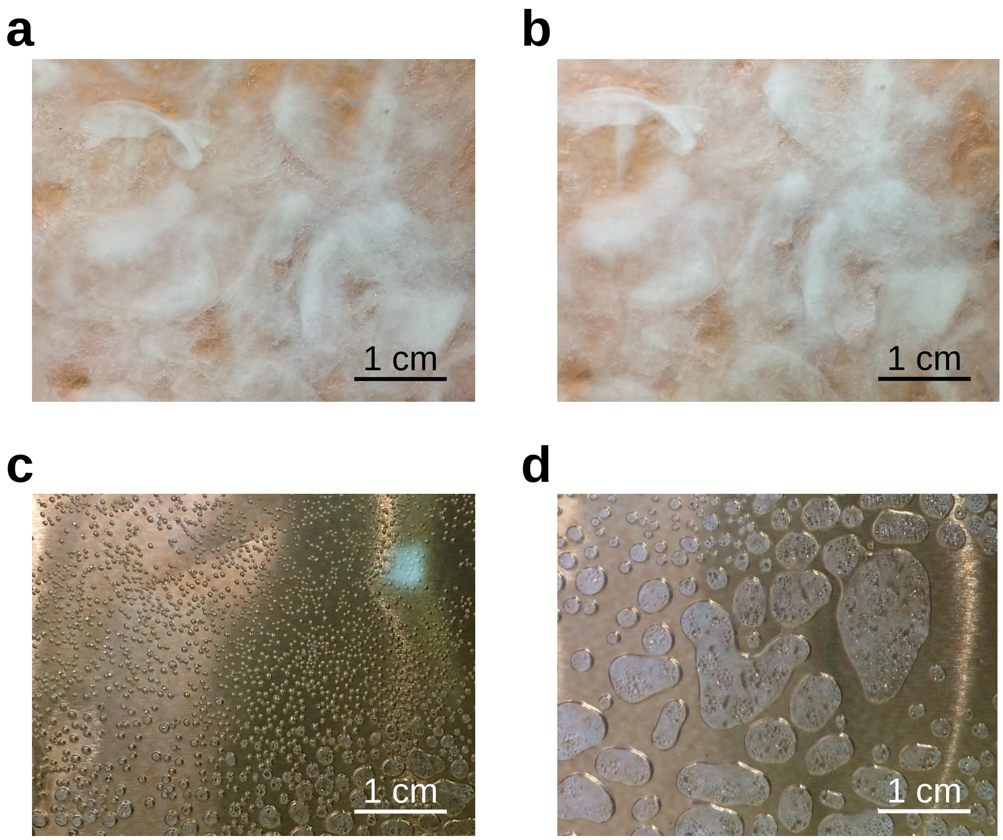


**Figure S5. Phase change of water in the narrow gap.** (**a**,**b**) Phase change of water confined in a narrow gap made of glass and copper surfaces and filled with hydrophilic cotton. Pictures are taken at different instants. (**c**) Phase change of water confined in a narrow gap made of glass and copper surfaces. After an initial nucleate boiling behavior, (**d**) transition boiling is then established in the narrow gap. Note that the observed two-phase patterns may differ respect to the ones under operating conditions, since the removal of the thermal insulating material from the top side of the glass layer causes a slightly different temperature distribution through the narrow gap.

## References


1. Ghasemi, H. *et al.* Solar steam generation by heat localization. *Nature communications* **5**, 4449 (2014).
2. Shah, M. M. Calculation of evaporation from indoor swimming pools: further development of formulas. *Ashrae Transactions* **118**, 460 (2012).
3. Ni, G. *et al.* Volumetric solar heating of nanofluids for direct vapor generation. *Nano Energy* **17**, 290–301 (2015).
4. Zhou, L. *et al.* Self-assembly of highly efficient, broadband plasmonic absorbers for solar steam generation. *Science advances* **2**, e1501227 (2016).
5. Ni, G. *et al.* Steam generation under one sun enabled by a floating structure with thermal concentration. *Nature Energy* **1**, 16126 (2016).
6. Liu, Z. *et al.* Extremely cost-effective and efficient solar vapor generation under nonconcentrated illumination using thermally isolated black paper. *Global Challenges* (2017).
7. Shirazy, M. R., Blais, S. & Fréchette, L. G. Mechanism of wettability transition in copper metal foams: From superhydrophilic to hydrophobic. *Applied Surface Science* **258**, 6416–6424 (2012).
8. Hu, H., Lai, Z., Ding, G., Zhuang, D. & Weng, X. Experimental investigation on water drainage characteristics of open-cell metal foams with different wettabilities. *International Journal of Refrigeration* **79**, 101–113 (2017).
